# Non-genetic adaptive resistance to KRAS^G12C^ inhibition: EMT is not the only culprit

**DOI:** 10.3389/fonc.2022.1004669

**Published:** 2022-11-22

**Authors:** Wenjuan Ning, Thomas M. Marti, Patrick Dorn, Ren-Wang Peng

**Affiliations:** ^1^ Division of General Thoracic Surgery, Inselspital, Bern University Hospital, University of Bern, Bern, Switzerland; ^2^ Department for BioMedical Research (DBMR), University of Bern, Bern, Switzerland

**Keywords:** non-genetic adaptive resistance, KRAS G12C inhibitors, EMT, symbiosis, TME

## Abstract

Adaptions to therapeutic pressures exerted on cancer cells enable malignant progression of the tumor, culminating in escape from programmed cell death and development of resistant diseases. A common form of cancer adaptation is non-genetic alterations that exploit mechanisms already present in cancer cells and do not require genetic modifications that can also lead to resistance mechanisms. Epithelial-to-mesenchymal transition (EMT) is one of the most prevalent mechanisms of adaptive drug resistance and resulting cancer treatment failure, driven by epigenetic reprogramming and EMT-specific transcription factors. A recent breakthrough in cancer treatment is the development of KRAS^G12C^ inhibitors, which herald a new era of therapy by knocking out a unique substitution of an oncogenic driver. However, these highly selective agents targeting KRAS^G12C^, such as FDA-approved sotorasib (AMG510) and adagrasib (MRTX849), inevitably encounter multiple mechanisms of drug resistance. In addition to EMT, cancer cells can hijack or rewire the sophisticated signaling networks that physiologically control cell proliferation, growth, and differentiation to promote malignant cancer cell phenotypes, suggesting that inhibition of multiple interconnected signaling pathways may be required to block tumor progression on KRAS^G12C^ inhibitor therapy. Furthermore, the tumor microenvironment (TME) of cancer cells, such as tumor-infiltrating lymphocytes (TILs), contribute significantly to immune escape and tumor progression, suggesting a therapeutic approach that targets not only cancer cells but also the TME. Deciphering and targeting cancer adaptions promises mechanistic insights into tumor pathobiology and improved clinical management of KRAS^G12C^-mutant cancer. This review presents recent advances in non-genetic adaptations leading to resistance to KRAS^G12C^ inhibitors, with a focus on oncogenic pathway rewiring, TME, and EMT.

## Introduction

Lung cancer is the most commonly diagnosed malignancies and the leading cause of cancer death worldwide, with 5-year survival rates still below 15% (1). The majority of patients with lung cancer are diagnosed with non-small cell lung cancer (NSCLC), which has benefited significantly from biomarker-guided targeted therapies (2). For example, EGFR tyrosine kinase inhibitors (e.g., gefitinib, erlotinib, and afatinib) and ALK tyrosine kinase inhibitors (e.g., crizotinib, ceritinib) have demonstrated superior objective response rates and significant better progression-free survival in NSCLC patients harboring epidermal growth factor receptor (EGFR) mutations or anaplastic lymphoma kinase (ALK) rearrangements than conventional one-fit-all chemotherapy (3, 4).

KRAS is the most frequently mutated oncoprotein in human cancers, affecting 25% to 30% of patients with NSCLC (5). Ironically, unlike the oncoproteins EGFR and ALK, which are less prevalently altered in NSCLC, there are few targeted therapies for *KRAS*-mutant NSCLC, and few clinical studies have specifically addressed this largest NSCLC subpopulation (6, 7). To date, direct inhibition of various mutant KRAS proteins has been a clinical challenge (7). Farnesyl transferase inhibitors designed to specifically inhibit KRAS by disrupting the protein’s association with the plasma membrane, showed little clinical efficacy, as did agents targeting effector proteins downstream of KRAS, such as the coveted RAF-MEK-ERK (MAPK) signaling pathway (8, 9).

The revolution in the fighting against KRAS-mutant cancers occurred in 2012 when a breakthrough study showed that KRAS with G12C (glycine to cysteine) substitution can be targeted by a group of small molecules that bind covalently to the substituted cysteine in the Switch-II pocket of the protein (10) (**Table 1**). This finding provided the impetus for further studies that eventually culminated in the FDA approval of the first KRAS inhibitor, sotorasib (AMG510), for the treatment of locally advanced or metastatic lung cancer with KRAS^G12C^ mutation, putting an end to the legend of “undruggable RAS proteins” (11). Since these inhibitors preferentially target GDP-bound KRAS (inactive form), a prerequisite for their efficacy is that KRAS^G12C^ retains GTPase activity, which converts the allosteric switch of KRAS^G12C^ from a GTP-bound to a GDP-bound conformation with assistance of GTPase-activating proteins (GAPs) such as neurofibromin 1 (NF1) (12, 13).

**Table 1:** KRAS^G12C^ inhibitors.

KRAS^G12C^ inhibitor	Chemical name	Drug name	Trade name(s)
1st generation	ARS853	–	–
2nd generation	ARS1620	–	–
In clinical trial	AMG510	Sotorasib	Lumakras, Lumykras
	MRTX849	Adagrasib	–
	HBI-2438	–	–
	JAB-21822	–	–
	JDQ443	–	–
	D-1553	–	–
	HS-10370	–	–

Despite this milestone, there is still an unmet need to target other KRAS-mutant alleles (e.g., G12D, G12V, G13D, and Q61H). In addition, KRAS^G12C^ inhibitors are confronted with low response rates (intrinsic resistance) and development of resistant disease (acquired resistance) (14–17). While intrinsic resistance occurs due to preexisting clonal cancer cells that are refractory to and outgrow upon treatment, cancer cells can also develop the phenotype of adaptive or acquired resistance during treatment. The general concept of intrinsic and acquired resistance to anticancer therapy Has been very recently reviewed elsewhere (18).

Cancer cells can develop drug resistance by acquiring novel genetic alterations that promote tumor growth, such as a novel missense mutation of the KRAS protein other than KRAS^G12C^ or at a site that affects the Switch-II pocket (S-IIP) conformation, or amplification of upstream receptor tyrosine kinases (RTKs) (19). Here, we focus on the mechanisms of resistance to KRAS^G12C^ inhibitor therapy driven by phenotypic plasticity and the identification of alternative strategies to overcome resistance. First, cancer cells can use non-genetic adaptions to counteract targeted inhibition of KRAS^G12C^ because oncogenic pathways are woven into intricate signaling circuits, allowing alternative pathways to assume the role of maintaining proliferating activities upon the inhibition of one pathway. Second, the tumor microenvironment (TME) of cancer cells, such as tumor-infiltrating lymphocytes (TILs), contribute significantly to immune escape and tumor progression, suggesting a therapeutic approach that targets not only cancer cells but also the TME. Third, EMT, an important phenotypic plasticity program, has been identified as a major cause of both intrinsic and acquired resistance to KRAS^G12C^ inhibitors, as well as inhibition of the MAPK pathway (20–22). This type of adaption take advantage of mechanism already present in cancer cells and does not require genetic modifications.

Recent evidence has shown that cancer cells can employ multiple mechanisms driven by non-genetic adaptations to counteract therapeutic pressure. Fully deciphering these mechanisms will provide new approaches to prevent cancer cells from escaping programmed cell death and to restore their susceptibility to KRAS^G12C^-targeted therapy (23). Interestingly, the adaptive response of cells to cancer therapy has in part in common with the phenotypic plasticity by which cancer cells evolve during metastasis (reviewed in (24)). In this context, it has been proposed that the biological pathways underlying the phenotypic plasticity of scattered tumor cells during metastasis can be classified into five categories, e.g., EMT, stemness, metabolism, dormancy, and host-organ mimicry (25). In this review, we extend this concept of phenotypic plasticity and specifically addresses therapy-induced plasticity of cancer cells (e.g., rewiring of oncogenic signaling pathways, phenotypic switching, and remodeling of the TME) in the context of resistance to KRAS^G12C^ inhibitors. In particular, we focus on the causal contribution of oncogenic signaling bypass, the symbiotic interaction between cancer cells and TME and EMT, and strategies to improve KRAS^G12C^ inhibitor therapy.

## Non-genetic adaptive resistance to kras^G12C^ inhibition: bypassing oncogenic signaling pathways

**Figure 1:**
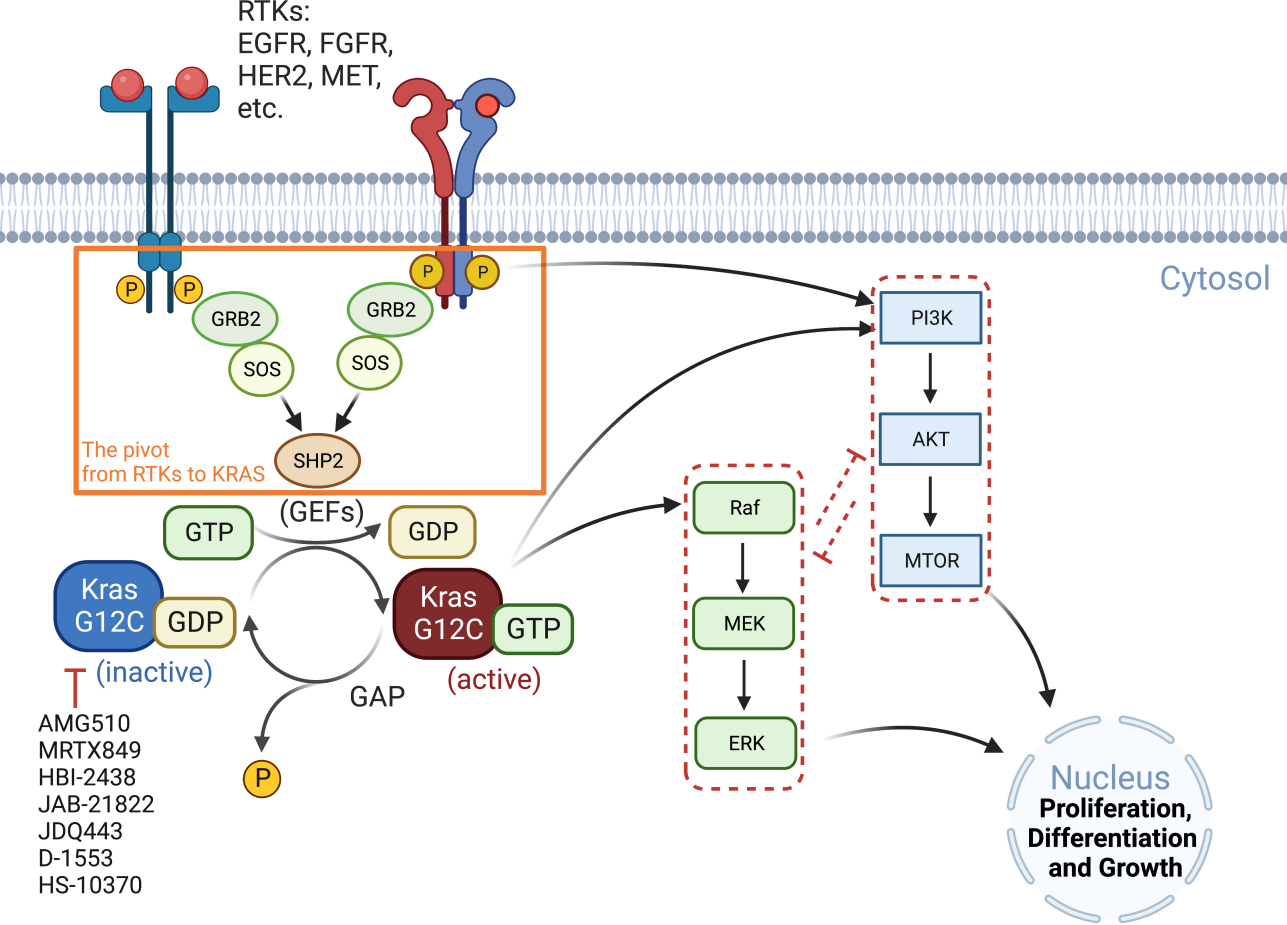
Oncogenic KRAS signaling pathway. KRAS switches between the GDP-bound inactive form and the GTP-bound active state, which is facilitated by GEFs and GAP, respectively. Activated RTKs relay extracellular signals from GRB2 to SOS, one of the major GEFs, to SHP2 and to KRAS. KRAS^G12C^ inhibitors (AMG510, MRTX849, etc.) preferentially target the GDP-bound inactive form of the KRAS protein and prevent its conversion to the active form (GTP-bound). The major signaling cascades upstream and downstream of KRAS are also highlighted. GAP, GTPase-activating protein; GEFs, guanine nucleotide exchange factors; GRB2, growth factor receptor-bound protein 2; P, phosphorylation; SHP2, Src homology region 2 domain-containing phosphatase-2; SOS, son of sevenless protein.

RAS proteins (KRAS, NRAS, and HRAS) transduce extracellular signals from upstream RTKs to downstream signaling pathways, with the mitogen-activated protein kinase (MAPK) cascade RAF-MEK-ERK and the PI3K-AKT-mTOR pathway being best studied (26). Although both pathways play critical roles in cell proliferation and survival, the MAPK pathway is considered the major downstream effector of RAS proteins (**Figure 1**).

The RAF-MEK-ERK and PI3K-AKT-mTOR pathways negatively interact with each other and thus may compensate when one of them is inhibited (27). Indeed, ARS1620, a second-generation covalent inhibitor of KRAS^G12C^, has been reported to synergize *in vitro* and *in vivo* with several PI3K inhibitors in KRAS^G12C^ mutant cancer cells (e.g., HCC44, H2122 and SW1573) that exhibit intrinsic resistance to ARS1620 (28). That RAF/MEK/ERK and PI3K/AKT/mTOR are tightly intertwined and compensate for each other has been further confirmed by the combinatorial effects of MEK and AKT inhibitors in RAS-mutated multiple myeloma, which significantly increased apoptotic cell death compared with single agents (29).

Inhibition of RAS-RAF-MEK-ERK signaling may also adaptively activate upstream RTKs by eliminating negative feedback loops, thereby activating other KRAS downstream effectors such as mTOR signaling and bypassing RAS-RAF-MEK-ERK, and promoting resistance to KRAS^G12C^ inhibitors. Indeed, it has been shown that ARS1620 downregulates the phosphorylation sites of EGFR that is inhibitory for EGFR activity. Moreover, ARS1620 could downregulate multiple inhibitory phosphorylation sites of HER2/3 and increased the total level of HER2/3 (30), suggesting that KRAS^G12C^ inhibition can abrogate EGFR/HER2/3 blockage and facilitate their activation. As a result, the combination of adagrasib (MRTX849) with EGFR or ERBB inhibitors was significantly better than single agents in xenograft models of KRAS^G12C^-mutant H2122 (NSCLC) and KYSE-410 (esophageal carcinoma) (31). Moreover, the anti-tumor efficacy of sotorasib is enhanced by the EGFR inhibitor cetuximab, as the drug combination significantly reduces cell viability *in vitro* and potently suppresses tumor growth in a patient-derived xenograft (PDX) model (32).

FGFR1 has also been reported to influence the response to KRAS^G12C^ inhibitors. In KRAS^G12C^ models, combined FGFR inhibitors with ARS1620 showed synergistic effects in mesenchymal subsets (30). MET, also known as hepatocyte growth factor receptor (HGFR), may play a similar role: it can activate RAS *via* GEFs. Independent of RAS, MET induces AKT activation, and its amplification has been shown to lead to AMG510 resistance in NSCLC cells. The combination of MET and KRAS^G12C^ inhibitors was able to limit tumor growth in xenograft models (33).

Inhibition of other nodes of the RAS-RAF-MEK-ERK axis also has the potential to increase the efficacy of KRAS^G12C^ inhibitors. A synergistic effect has been observed by dual inhibition of MEK and FGFR1 in genetically engineered mouse models, and an increase in FRS2, the FGFR adaptor protein, has been reported to promote KRAS^G12C^ inhibitor resistance (9, 34). Combined inhibition of BRAF and EGFR effectively improves the response of BRAF(V600E) colon cancers to BRAF inhibitors (35). Upregulation of EGFR and platelet-derived growth factor receptor (PDGFRβ) by TGF-β signaling leads to resistance to BRAF and MEK inhibitors (36), and upregulation of PDGFRα by the Sonic Hedgehog Homolog (Shh) pathway confers resistance to BRAF inhibition in metastatic BRAF(V600E) melanoma (37). Similarly, co-targeting MEK and SHP2 intensively blocks RTK-RAS signaling and is superior to inhibiting individual RTKs as RTKs phosphorylate and activate SHP2 and promote signaling from SOS1/2 to RAS (38).

KRAS^G12C^ inhibitors bind to the GDP-bound inactive KRAS protein, so upstream signaling molecules that promote the allosteric switch from the inactive to the active conformation of the protein also promote resistance to KRAS^G12C^ inhibitors. SOS1 is a guanine nucleotide exchange factor (GEF) that activates RAS, and SHP2 (SH2 containing protein tyrosine phosphatase-2) is a tyrosine phosphatase that activates SOS1-regulated RAS-GTP loading. As an overlapped node in RTKs to RAS cycle, it is not surprisingly that these factors are now being targeted as a new therapeutic framework, with improved anti-tumor efficacy observed by co-targeting SHP2 and KRAS^G12C^, regardless of ARS1620, AMG510, or MRTX849 (19, 39, 40).

Several novel signaling pathways have been shown to compensate for KRAS signaling. Polo‐like kinase 1 (PLK1) is a serine/threonine kinase with pleiotropic functions in mitosis and in response to DNA damages by regulating ataxia-telangiectasia mutated (ATM) and ATM- and Rad3-Related (ATR) checkpoint activity. Inhibition of PLK1 leads to synthetic lethality in RAS-mutant cells because RAS mutations are associated with mitotic stress, rendering RAS-mutant cells more dependent upon on PLK1 activity for proper mitotic progression (41). We have recently shown that dual inhibition of PLK1 and FGFR1 has synergistic anticancer effects in KRAS-mutant cancer cells, as FGFR1 and PLK1 cooperate control the metabolic stress associated with KRAS mutation (42). We summarize recently identified targets and strategies that improve KRAS^G12C^ inhibitor therapy in **Table 2**.

**Table 2:** Targets and strategies to improve the efficacy of KRAS^G12C^ inhibitors.

RAS signaling nodes		Combination target	Reference
KRAS-G12C		PI3K/AKT/mTOR	Misale, S., et al. (28)
		SHP2/SOS	Lou, K., et al. (40), Fedele, C., et al. (39), Hallin, J., et al. (31), Solanki, H.S., et al. (30)
		EGFR	Hallin, J., et al. (31), Amodio, V., et al. (32)
		HER2/HER3	Solanki, H.S., et al.( 30), Ho, C.S.L., et al.(2021)
		FGFR	Solanki, H.S., et al.( 30)
		MET	Suzuki, S., et al. (33)
MAPK	BRAF MEK	EGFR	Prahallad, A., et al. (35)
		PDGFRα/PDGFRβ	Sun, C., et al. (36), Sabbatino, F., et al. (37)
		FGFR	Manchado, E., et al. (9), Lu, H., et al. (34)
		SHP2/SOS	Fedele, C., et al. (38)

## Non-genetic adaptive resistance kras^G12C^ inhibition: symbiosis of cancer cells with the TME

**Figure 2:**
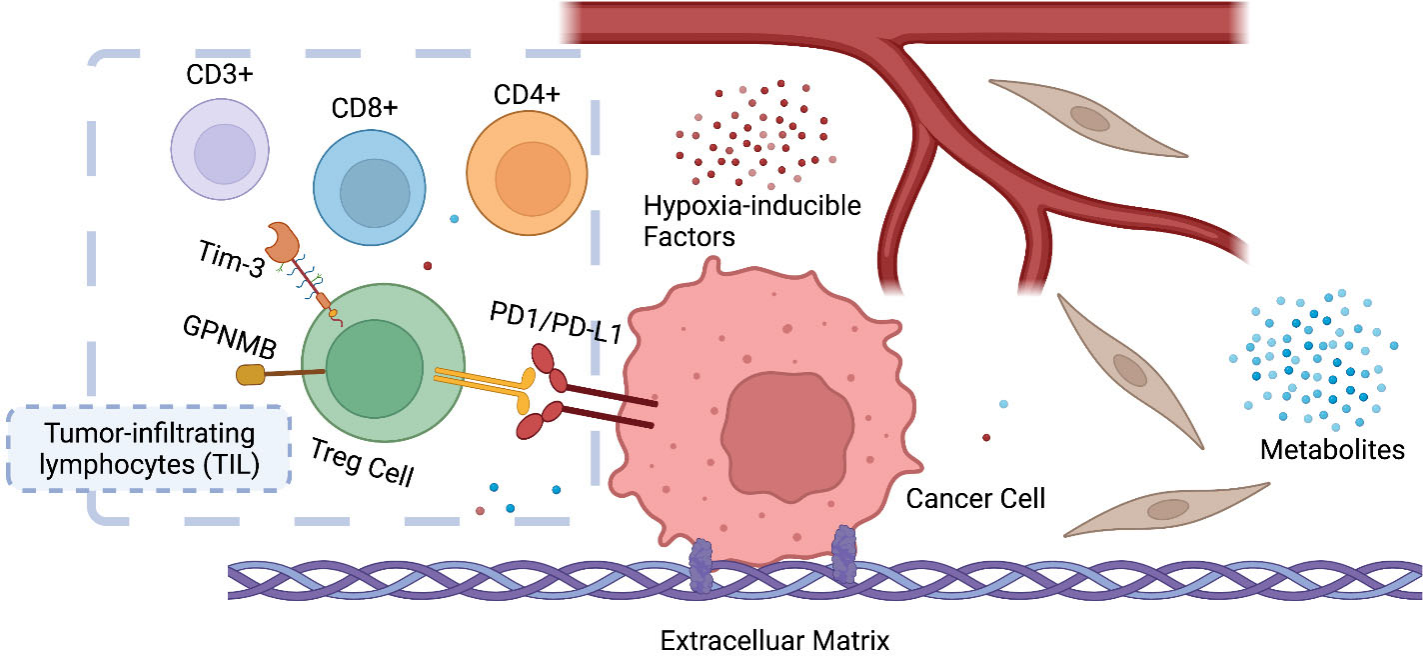
Symbiosis between cancer cells and the tumor microenvironment (TME). The infiltration and ratio of different lymphocytes are determined by the antigen presentation of cancer cells, which in return influences tumor growth and response to therapy. Hypoxia-inducible factors (HIF) and metabolites (e.g., lactate) also play a key role in reprogramming the TME of cancer. CD3^+^ T-lymphocyte: T cells that mediate the activation of tumor-reactive T cells, e.g., CD8^+^ naive T cells and CD4^+^ naive T cells. CD4^+^ T lymphocyte: also called T helper cell, which remodels TME by releasing cytokines and mediates the anti-tumor response of CD8^+^ T cells by cross-presentation of dendritic cells. CD8^+^ T lymphocytes: also called cytotoxic T cell, the specific killer that targets the surveilled cancer cells. Treg cells: also called suppressor T cells, a subpopulation of T cells that modulate the immune system, maintain tolerance to self-antigens, and prevent autoimmune disease. Treg cells are immunosuppressive and generally suppress or downregulate the induction and proliferation of effector T cells. Treg cells express CD4, FOXP3, and CD25 and are thought to be derived from the same lineage as naïve CD4^+^ cells. Since effector T cells also express CD4 and CD25, it is difficult to effectively distinguish Treg cells from effector CD4^+^ cells, making them difficult to study.

The tumor microenvironment (TME), the niche surrounding the cancer cells, consists of normal resident cells, immune cells, fibroblasts, stromal cells, blood vessels, signaling molecules, metabolites, and the extracellular matrix (ECM). Tumor and the TME co-exist as a symbiotic unit and constantly interact, which plays a critical role in defense against external stimuli such as anticancer drugs (**Figure 2**). Tumor cells even recruit immune cells as “partners in crime”. Although the mechanisms underlying immune escape are not fully understood, it has been shown that tissue-resident macrophages protect cancer cells from immune surveillance by upregulating regulatory T-cell (Treg) responses (43).

Remodeling TME significantly affects tumor response to anticancer drugs, which involves not only immune cells but also other symbiotic components such as coagulation and angiogenesis. RAS/PI3K promotes the expression of angiogenic factors, e.g., vascular endothelial growth factor A (VEGFA), *via* cyclooxygenase 2 (COX2) (44) and activation of tumor angiogenesis and coagulation pathways leads to adaption to sotorasib (45). Consequently, COX2 inhibition *via* PI3K impairs anti-angiogenesis.

The programmed death-1 (PD-1)/PD-1 ligand 1 (PD-L1) axis expressed on activated T cells and cancer cells functions as an immune checkpoint. The interaction of PD-L1 with PD-1 silences the T cells, resulting in so-called tumor-induced immunosuppression (46). PD-1/PD-L1 inhibitors prevent the interaction, reactivate T cell function, and kill cancer cells. Other immune checkpoints such as T-cell immunoglobulin mucin-3 (Tim-3) and transmembrane glycoprotein NMB (GPNMB), increase sharply after PD-1/PD-L1 blockade, and inhibition of Tim-3 or GPNMB can reverse anti-PD-1 treatment failure (47, 48). After 24-h exposure to an anti-PD-1 antibody (10 μg/ml) on tumor-infiltrating lymphocytes (TILs), Tim-3 expression was increased by 50% and 40% in CD8^+^ T cells and in CD4^+^CD25^low/−^ effector T cells, respectively (49). It was reported that Tim-3 activation is mediated by PI3K/AKT/mTOR, which plays a key role in inflammatory response (50), and that SHP2 inhibition increases the ratio of CD8^+^/Treg cells and sensitize tumors to PD-1 inhibition in pancreatic ductal adenocarcinoma (PDAC) and NSCLC models (39).

In a syngeneic KRAS^G12C^ colon cancer model, the number of total and proliferating CD3^+^ T cells as well as CD8^+^ T cells increased after AMG510 treatment, suggesting remodeling of the TME by AMG510. AMG510 plus PD-1 inhibitors resulted in long-term tumor-specific T cell responses (51, 52). However, a reduction of adaptive immune responses was also observed in sotorasib-resistant tumors, and immune escape may be a crucial factor contributing to KRAS^G12C^ inhibition resistance (45).

## Non-genetic adaptive resistance kras^G12C^ inhibition: EMT and other transcriptional/post-transcriptional adaptions

**Figure 3:**
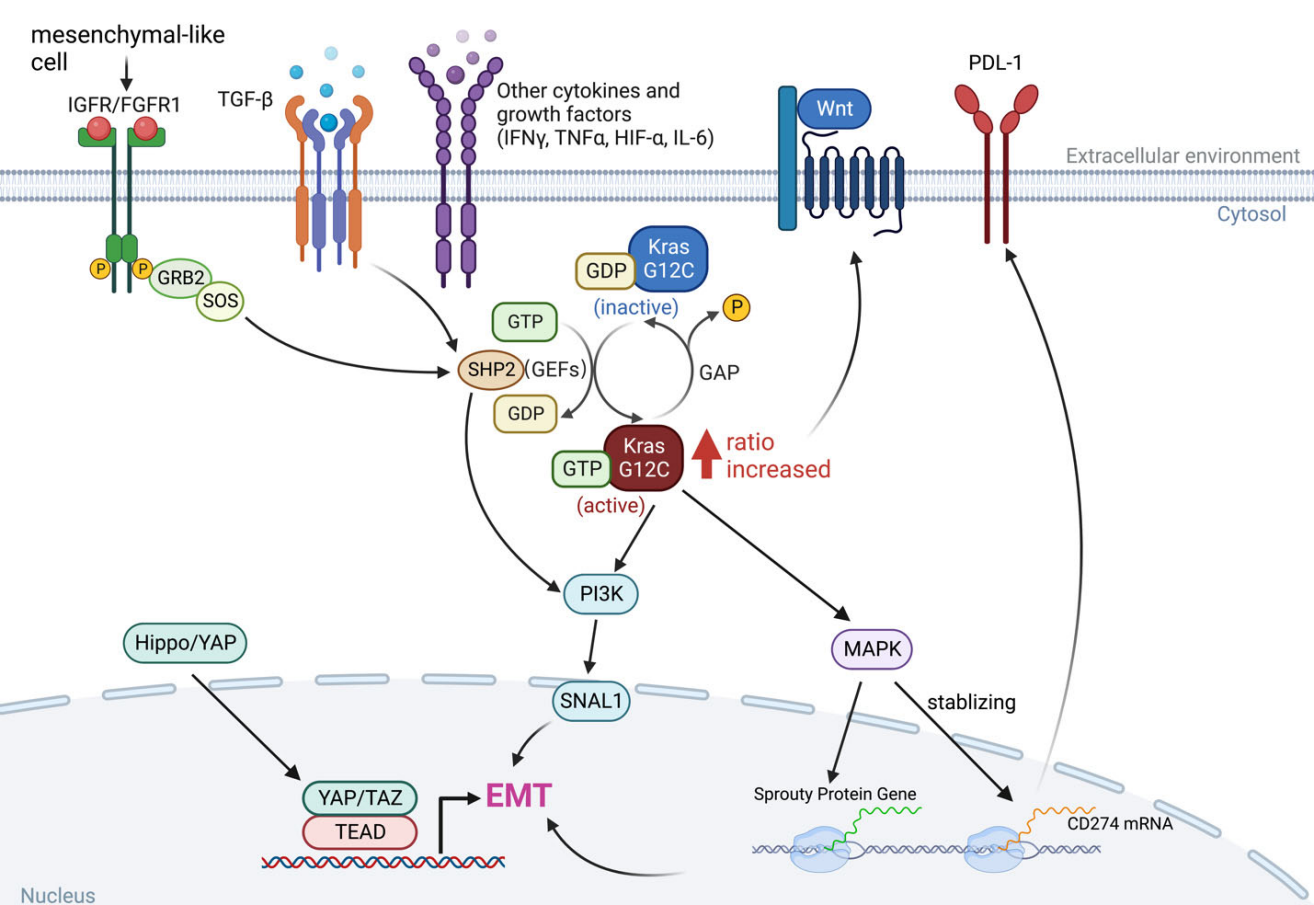
The interaction between KRAS signaling and EMT. The KRAS-MAPK pathway is important for the stability of CD274 (PD-L1) mRNAs. KRAS signaling and YAP/TAZ converge to activate transcriptional programs that regulates EMT and EMT is a key driver of tumor immune evasion. IGFR, insulin-like growth factor receptor; FGFR1, fibroblast growth factor receptor 1; TGF-β, transforming growth factor-beta; IFN-γ, interferon gamma; TNF-α, tumor necrosis factor-alpha; HIF-α, hypoxia-inducible factor-alpha; IL-6, interleukins 6; SNAI1, snail family transcriptional repressor 1.

Epithelial-to-mesenchymal transition (EMT) is the manifestation of a series of epigenetic and biochemical alterations that enable the phenotypic change from an epithelial to a mesenchymal cell phenotype (53). A variety of biochemical drivers can lead to this progression, e.g., transforming growth factor-beta (TGF-β), tumor necrosis factor-alpha (TNF-α), hypoxia-inducible factor-alpha (HIF-α), Wnt signaling, Interleukins (IL-1β, IL-6), Hedgehog, and the Hippo pathway (54–56), and impart cancer cells with properties of mesenchymal stem cells, drug resistance and invasiveness (**Figure 3**).

Long-term exposure to TGF-β increased the ratio of GTP-bound KRAS protein level in KRAS^G12C^ mutant malignancies, as did in Twist- or Snail-expressing mesenchymal cells. In KRAS^G12C^ mutant cancers, the amount of GTP-bound KRAS proteins determines the sensitivity to KRAS^G12C^ inhibitors, which interacts with and blocks KRAS^G12C^ when it is in the inactive GDP-bound state (**Figure 1**), so an increased ratio of KRAS-GTP versus KRAS-GDP cause resistance to KRAS^G12C^ inhibitors (20, 57).

Regardless of inhibiting KRAS itself or the downstream MAPK pathway, EMT is blameworthy for drug resistance (45). Activation of the PI3K pathway in mesenchymal-like KRAS^G12C^ mutant cancer cells could be the molecular basis for EMT-mediated resistance or, alternatively, could be due to a cell cycle alteration leading to CDK4-dependent growth (58). Cells expressing high levels of CSNK2A1 (Casein Kinase 2 Alpha 1) were found to have an increased mesenchymal gene signature, and reduction of CSNK2A1 converted the cells to the epithelial type and restored their sensitivity to KRAS^G12C^ or MEK inhibitors (59). Therefore, strategies that promote mesenchymal-to-epithelial transition (MET) are promising to overcome resistance to KRAS^G12C^ inhibitors.

The KRAS-MAPK axis has been shown to be associated with immune checkpoint activity through a mechanism that controls the post-transcriptional functions of immune checkpoint proteins. PD-L1 is encoded by *CD274* and MAPK signaling has been shown to play a critical role in stabilizing *CD274* mRNA, increasing PD-L1 protein levels and consequently promoting peripheral immune tolerance (60). As a result, inhibition of the RAS-MAPK pathway prevents EGF- and IFNγ-induced PD-L1 expression by suppressing *CD274* mRNA and augments the efficacy of immunotherapy (51, 52, 61). More importantly, tumor cells undergoing EMT can escape immune surveillance, suggesting that EMT is involved in the acquisition of resistance to immunotherapy (62). Indeed, Snail has been associated with the induction of immunosuppressive cytokines, activation of regulatory T cells (Treg), and the generation of impaired dendritic cells (63). EMT in tumor cells that have undergone phenotypic changes has significant effects on the recognition of cancer cells by the native and adaptive immune systems. Both down- and up-regulation of cell surface molecules with immunological significance have been described (64). In general, these changes are accompanied by immune resistance and evasion, although exceptions to this rule have also been reported (**Figure 3**).

Yes-associated protein (YAP) and TEA domain 2 (TEAD2) are a transcriptional co-regulator and a downstream effector of Hippo signaling pathway, respectively, that play critical roles in controlling the expression of several EMT-related genes and have been reported to confer resistance to multiple drugs (65, 66). The relationship between YAP and the RAF/MEK/ERK cascade was discovered by genetic screens, which showed that the inhibitory combination of RAF or MEK with YAP has increased efficacy not only in BRAF-mutant cancers but also in KRAS-mutant cancers (67). In a KRAS^G12C^ mutant PDAC model, inhibition of YAP1 improves the efficacy of KRAS blockade (68).

c-MYC is another oncogenic transcription factor being involved in crucial processes such as metabolic reprograming, extracellular matrix remodeling, inflammation, and regulation of a variety of malignant features in cancer (69). KRAS controls c-MYC by stabilizing the protein stability and activation of c-MYC in turn promotes KRAS-driven oncogenic potential. For example, KRAS^G12C^ promotes cap-dependent translation initiation and c-MYC is an indirect indicator of the process (70). Further, KRAS and c-MYC cooperate to drive an immunosuppressive TME in cancer development, leading to increase in macrophage infiltration of tumours and decrease in CD3+ T cells, B cells and natural killer (NK) cells. These changes in the TME precede an increase in tumour size and are promoted by tumour cell-derived CC-chemokine ligand 9 (CCL9) and interleukin−23 (IL−23). Depletion of these cytokines can reduce tumour development as CCL9 is crucial for infiltration of macrophages, angiogenesis and T cell loss, and IL−23 is crucial for loss of T, B and NK cells. Infiltrating macrophages also express PD-L1, which is required for loss of T cells. Consequently, Myc deactivation rapidly reverse the observed stromal changes and induce tumour cell apoptosis and NK cell-dependent regression of Kras-driven lung adenocarcinoma in mice (71).

Overexpression of c-MYC in cancers leads to extracellular matrix (ECM) degradation and promotes angiogenesis, which in turn contributes to malignant invasion and metastasis. Overall, deregulation of c-MYC not only drives an oncogenic signaling in cancer cells, but also impinges on the TME by linking cellular signaling pathways, EMT, and the TME (72, 73). Thus, it is not surprising that amplification of the MYC gene results in drug resistance to KRAS^G12C^ inhibition (70).

## Conclusion

The development of covalent inhibitors that effectively and selectively target KRAS^G12C^ represents an unprecedented breakthrough in the personalized treatment of patients with KRAS-mutant cancers. This advance has ushered in a new era of targeted therapy that distinguishes the G12C mutation from other KRAS mutations (e.g., G12D, G12S, G12V, Q61H), resulting in selective eradication of KRAS^G12C^-mediated oncogenic signaling without affecting other KRAS substitutions and normal tissues. However, the perennial problem of resistance to targeted therapies also apply here, pointing to the pressing need to explore and therapeutically exploit the underlying mechanisms to overcome resistance to and maximize the efficacy of KRAS^G12C^ inhibitor therapy.

Current evidence suggests a multifaceted mechanism of resistance to KRAS^G12C^ inhibitor therapy that involves both tumor-intrinsic and -extrinsic processes. In addition to resistance mechanisms driven by genetic alterations in cancer cells, non-genetic adaptations mediated by rewiring of oncogenic signaling pathways, reciprocal interactions between cancer cells and TME, and phenotypic plasticity such as EMT are among the key strategies used by cancer cells to acquire a stem cell phenotype, an immunosuppressive niche, and, in particular, drug resistance.

Because the central role of KRAS is mediated by diverse cellular processes that not only occur in cancer cells but also involve the TME, this versatility of KRAS effector pathways is destined to dictate diverse adaptions that can be undertaken under treatment pressure. A comprehensive and in-depth understanding of resistance mechanisms will ultimately and profoundly transform the therapeutic landscape of KRAS^G12C^ inhibitors, although neither a universal solution nor limited versatility of mode of action is likely. This underscores the heterogeneity of KRAS^G12C^-mutant tumors and the need to consider other factors, such as genetic alterations co-occurring with KRAS^G12C^ that contribute to drug resistance, in developing precision medicine. Combination therapy holds the potential to increase efficacy and selectivity, reduce single-drug dosing, decrease the development of drug resistance, and possibly avoid toxicity, and thus has emerged as an effective strategy for the treatment of refractory cancers. Nevertheless, the advent of potent and selective inhibitors for KRAS^G12C^ is definitely not the beginning of the end, but the end of the beginning for the era of precision medicine, as this breakthrough has spurred the search for mutation-specific targeted therapies, as evidenced by the most recent development of KRAS^G12D^ inhibitors (74).

## Author contributions

WN reviewed existing literature, prepared the figure and table, and wrote the manuscript; TM and PD edited and revised the manuscript; R-WP conceived the study, edited, and revised the manuscript; All authors contributed to the article and approved the submitted version.

## Funding

This study was supported by a grant from Swiss National Science Foundation (SNSF #310030_192648; to R-WP). WN is supported by a PhD fellowship from China Scholarship Council (WN).

## Conflict of interest

The authors declare that the research was conducted in the absence of any commercial or financial relationships that could be construed as a potential conflict of interest.

## Publisher’s note

All claims expressed in this article are solely those of the authors and do not necessarily represent those of their affiliated organizations, or those of the publisher, the editors and the reviewers. Any product that may be evaluated in this article, or claim that may be made by its manufacturer, is not guaranteed or endorsed by the publisher.
